# A probabilistic molecular fingerprint for big data settings

**DOI:** 10.1186/s13321-018-0321-8

**Published:** 2018-12-18

**Authors:** Daniel Probst, Jean-Louis Reymond

**Affiliations:** Department of Chemistry and Biochemistry, National Center for Competence in Research NCCR TransCure, University of Berne, Freiestrasse 3, 3012 Bern, Switzerland

**Keywords:** Virtual screening, Similarity search, Fingerprints, Locality sensitive hashing, Approximate k-nearest neighbor search

## Abstract

**Background:**

Among the various molecular fingerprints available to describe small organic molecules, extended connectivity fingerprint, up to four bonds (ECFP4) performs best in benchmarking drug analog recovery studies as it encodes substructures with a high level of detail. Unfortunately, ECFP4 requires high dimensional representations (≥ 1024D) to perform well, resulting in ECFP4 nearest neighbor searches in very large databases such as GDB, PubChem or ZINC to perform very slowly due to the curse of dimensionality.

**Results:**

Herein we report a new fingerprint, called MinHash fingerprint, up to six bonds (MHFP6), which encodes detailed substructures using the extended connectivity principle of ECFP in a fundamentally different manner, increasing the performance of exact nearest neighbor searches in benchmarking studies and enabling the application of locality sensitive hashing (LSH) approximate nearest neighbor search algorithms. To describe a molecule, MHFP6 extracts the SMILES of all circular substructures around each atom up to a diameter of six bonds and applies the MinHash method to the resulting set. MHFP6 outperforms ECFP4 in benchmarking analog recovery studies. By leveraging locality sensitive hashing, LSH approximate nearest neighbor search methods perform as well on unfolded MHFP6 as comparable methods do on folded ECFP4 fingerprints in terms of speed and relative recovery rate, while operating in very sparse and high-dimensional binary chemical space.

**Conclusion:**

MHFP6 is a new molecular fingerprint, encoding circular substructures, which outperforms ECFP4 for analog searches while allowing the direct application of locality sensitive hashing algorithms. It should be well suited for the analysis of large databases. The source code for MHFP6 is available on GitHub (https://github.com/reymond-group/mhfp).
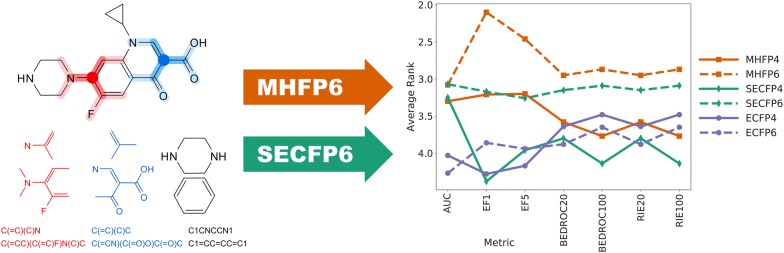

**Electronic supplementary material:**

The online version of this article (10.1186/s13321-018-0321-8) contains supplementary material, which is available to authorized users.

## Introduction

Many uses of cheminformatics require the quantification of the similarity between molecules. As the underlying data structure used to represent molecules is a graph, this problem is equivalent to a subgraph isomerism problem, which is at least NP-complete [1]. Molecular fingerprints reduce this problem to the comparison of vectors, enabling further application of approximation methods and heuristics, thus speeding up the computation [2–5].

Among the assortment of fingerprints for the comparison of molecules in use today, extended connectivity fingerprint (ECFP) is the most prominent due to its outstanding performance in molecular structure comparisons requiring the identification of compounds with similar bioactivity, as assessed in benchmarking studies [6, 7]. However, the performance of ECFP results from a precise encoding of molecular structure, which is achieved by using high-dimensional vectors, typically $d \ge 1024$, with the consequence that linear searching becomes slow when applied to very large databases such as GDB, PubChem or ZINC [8–10]. For more complex tasks such as constructing $k$-nearest neighbor graphs, linear search takes $O(dn^{2} )$ time, becoming prohibitively slow. This problem occurs even when applying commonly used optimized search algorithms such as k–d or ball trees, as well as algorithms from the R- and B-tree families, because their performance degrades to linear time due to the curse of dimensionality [11–13]. In addition, given the often binary, relatively sparse, and high dimensional nature of ECFP, $L^{p}$ metrics generally perform badly, further limiting the number of available optimization techniques. In the past, several approaches to remove the curse of dimensionality’s impact on nearest neighbor searching have been presented by the cheminformatics community. Most notably the BitBound method, which exploits simple bounds on similarity measures and indexing to achieve sub-linear speed on exact nearest neighbor searches with a time complexity of $O(n^{0.6} )$ for many metrics, including Jaccard similarity [14, 15]. In our effort to facilitate the exploration of very large databases such as GDB, we previously used lower dimensionality fingerprints such as MQN (Molecular Quantum Number, 42D) or SMIfp (SMILES fingerprint, 34D) for similarity searches, however, such fingerprints only encode molecular composition and do not allow precise structural similarity calculation [16–18].

**Fig. 1:**
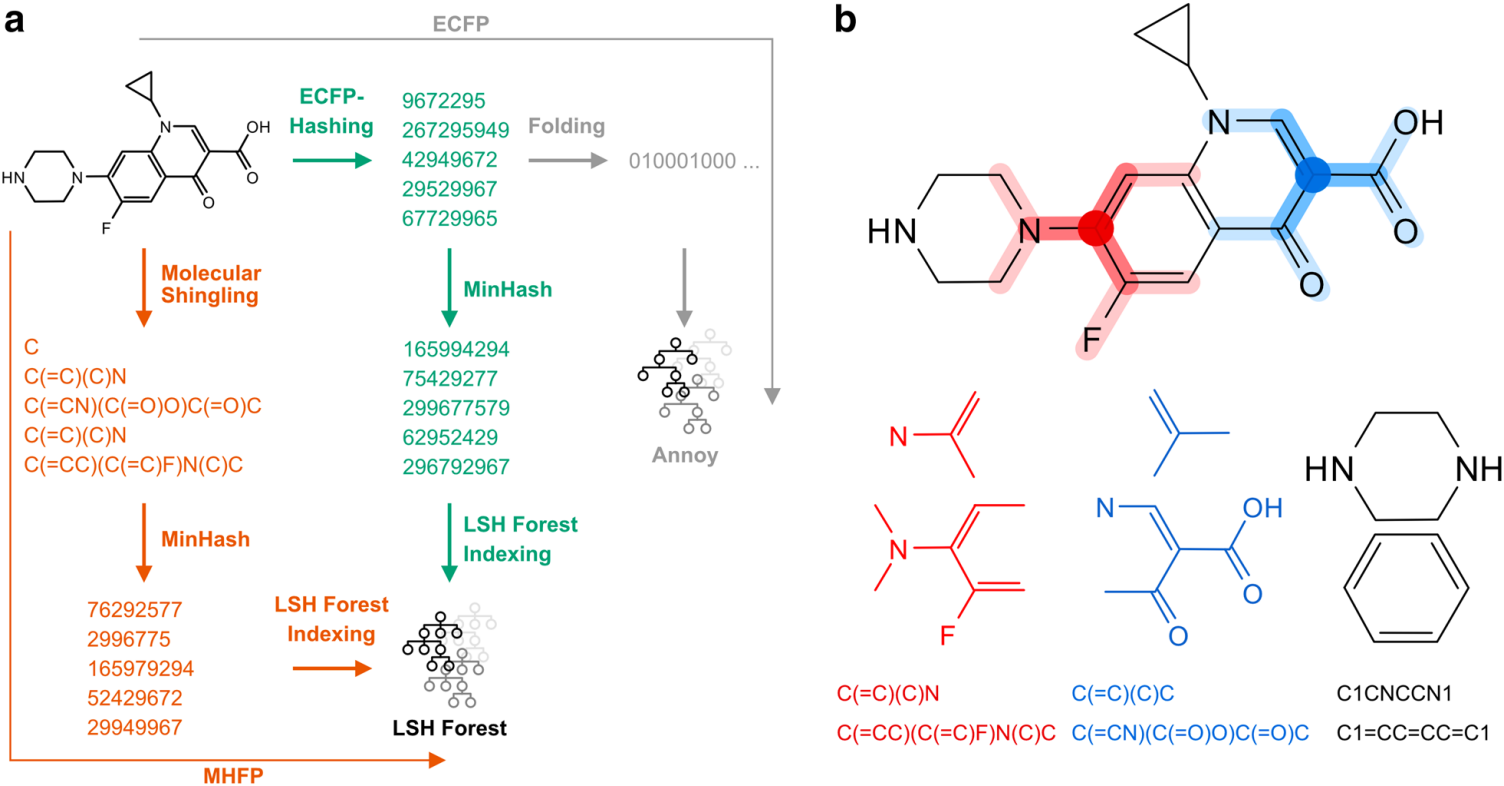
MHFP, ECFP workflow comparison. **a** Comparison of hashing and approximate nearest neighbor search indexing of ECFP with Annoy (gray) and MHFP via molecular shingling and MinHash with LSH Forest (orange). In addition, MinHash is applied to unfolded ECFP hashes and indexed using LSH Forest as well (green), resulting in the hybrid fingerprint MHECFP. The latter was used as a control to separate the influences of molecular shingling and applying MinHash on the measured performance. **b** Circular substructure SMILES of an input molecule are computed with each heavy atom as the center (examples for MHFP4 shown in red and blue). In addition, SMILES for each ring are extracted (examples shown in black). Circular substructure SMILES are rooted at the central atom. All substructure SMILES are canonicalized and kekulized

Herein we report a new family of fingerprints termed MHFP (MinHash fingerprint) which combine the circular nature of ECFP with w-shingling and MinHash, which are encoding and comparison methods used in natural language processing and text mining [19–21] (Fig. 1). These methods are commonly used in applications such as discarding already indexed web pages during web-crawling, signal processing or plagiarism detection [22, 23]. We obtain our MHFP by first writing out circular substructures around each atom as SMILES, a process which we call molecular shingling in analogy to the w-shingling scheme used for the above-mentioned text mining applications. We then apply the MinHash hashsing scheme to assign these SMILES to bit values in our MHFP.

MinHash is a locality sensitive hashing (LSH) scheme which applies a family of hashing functions to the substrings in a molecular shingling and stores the minimum hash generated from each hashing function in a set. These sets, containing the minimum hash values, have the interesting property that they can be indexed by an LSH algorithm for approximate nearest neighbor search (ANN), removing the curse of dimensionality [24]. While a previously reported LSH implementation for chemical structure indexing and searching was based on embeddings in Euclidean space, MinHash allows for the indexing of chemical structures in extremely sparse Jaccard (Tanimoto) space, a metric more appropriate for fingerprint-based similarity calculations [25, 26]. Note that LSH search algorithms cannot be directly applied to ECFP hashes due to the nature of the primary hashing scheme used to assign circular substructures to bit values. Furthermore, ECFP encodes circular substructures by iteratively hashing atomic invariants. Common implementations of ECFP, as found in RDKit or Open Babel, contain a default or hardcoded selection of atomic invariants to be hashed that is targeted towards applications in medicinal chemistry, thereby making assumptions regarding the importance of atomic features such as acidity or charge, thereby introducing a potential bias which is entirely avoided in MHFP, as it takes all information encoded in the SMILES into account [6, 27–29].

To assess the performance of MHFP we compare it to variants of ECFP as well as to a hybrid fingerprint MHECFP which applies MinHash to unfolded ECFP hashes. We find that the performance of MHFP surpasses that of ECFP and MHECFP in a ligand-based virtual screening benchmark [7]. Furthermore, MHFP allows for ANN searching using the LSH Forest algorithm, which enables the search of the very sparse and high-dimensional binary chemical space without folding, thus better preserving locality. MHFP6, encoding substructures up to a diameter of 6 bonds, performs best and should be considered as replacement for ECFP4 to improve searches in very large databases. The source code for MHFP is available on GitHub (https://github.com/reymond-group/mhfp).

## Methods

### Jaccard similarity

The Jaccard similarity is also referred to as Jaccard index, Jaccard similarity coefficient or Tanimoto index. Given two sets $A$ and $B$, the Jaccard similarity coefficient of the molecules is calculated as:

1 $$J\left( {A,B} \right) = \frac{{\left| {A \cap B} \right|}}{{\left| {A \cup B} \right|}}$$

The Jaccard distance is a metric defined as $1 - J\left( {A,B} \right)$ [30]. Both, the Jaccard similarity coefficient and distance have been shown to be appropriate for fingerprint-based similarity calculations [25].

### MinHash

MinHash is used to estimate the Jaccard similarity between two sets [19]. Given sets of integers, such as hash values, MinHash is applied as follows:

Let $a$ and $b$ be $k$-dimensional vectors with elements set to unique randomly generated integers such that $a_{i} ,b_{i} \in \left\{ {0, \ldots ,2^{32} - 1} \right\}$ and let $H$ be the set of all hash values $\left\{ {0, \ldots ,2^{32} - 1} \right\}$. Given a family of sets $F = \left\{ {S_{1} , \ldots S_{n} } \right\}$ over $H$ where each set represents a molecule, the MinHash function $h_{min} \left( {S_{i} ,a,b} \right)$ is applied to each set $S_{i}$ in $F$. Let $s$ be the vector form of a set $S$ from $F$ and $p$ be the Mersenne prime $2^{61} - 1$. The MinHash of a molecular graph is then calculated as:

2 $$h_{min} \left( {s_{i} ,a,b} \right) = min\left( {\left( {\left( {a \cdot s_{i}^{T} + b} \right) mod p} \right)mod\left( {2^{32} - 1} \right)} \right)$$

The set form $S_{min}$ of $s_{min}$ can then be used to estimate the Jaccard similarity coefficient of two sets $S_{i}$, $S_{j}$ using Eq. 1 [31].

The expected error of estimating the Jaccard similarity coefficient between two sets using MinHash is $O\left( {\frac{1}{{{ \log }\left( {\text{n}} \right)}}} \right)$, where $n$ is the number of hash functions used [32].

### LSH forest

The local sensitivity hashing (LSH) forest algorithm is an extension to LSH similarity indexing [33, 34]. Introducing self-tuning indices, the algorithm renders data-dependent manual parameter tuning superfluous by storing the hashes in multiple prefix-trees that make up the LSH Forest.

### Estimate number of hash collisions

As hash functions for strings are non-injective, so-called hash collisions occur when two or more non-identical strings are being hashed to an identical integer. The number of hash collisions can be estimated through a generalization of the birthday problem [35]:

3 $$c\left( {m,N} \right) = m - N\left( {1 - \left( {\frac{N - 1}{N}} \right)^{m} } \right)$$

where $m$ is the number of hashed values and $N$ is the maximum hash value.

### Annoy

Approximate Nearest Neighbors Oh Yeah (Annoy) is an approximate nearest neighbor searching library implemented by Spotify Technology S.A. to enable music recommendations [36]. While other distance metrics are available, the cosine distance is the metric supported by Annoy best suited for binary fingerprint indexing. The cosine similarity is defined as:

$$C\left( {A,B} \right) = \frac{{\mathop \sum \nolimits_{i = 1}^{n} A_{i} B_{i} }}{{\sqrt {\mathop \sum \nolimits_{i = 1}^{n} A_{i}^{2} } \sqrt {\mathop \sum \nolimits_{i = 1}^{n} B_{i}^{2} } }}$$

The cosine distance is, analogous to the Jaccard distance, defined as $1 - C\left( {A,B} \right)$.

### Statistical methods

The confidence level α is 0.05 for both the independent (unpaired) $t$-tests and the pairwise post hoc Friedman tests. The independent (unpaired) $t$-tests are computed using SciPy (1.1.0), the pairwise post hoc Friedman tests are part of the open-source platform to benchmark fingerprints for ligand-based virtual screening [7].

### Python implementation

The methods for generating molecular shinglings and computing the MinHash values described above were implemented in a Python (3.6.3) script that takes a SMILES string as an input and returns a NumPy (1.15.1) array of hashes, describing the molecule [37]. The cheminformatics library RDKit (2017_09_1) was used to parse the SMILES and extract substructures form the molecular graph (Fig. 1b) [27]. In order to evaluate the performance of MHFP in combination with LSH-based methods, a Python script implementing the locality sensitive hashing (LSH) forest algorithm for $k$-nearest neighbor searching according to the datasketch Python library was written [33, 38, 39]. The LSH Forest script returns the approximate $k$-nearest neighbors of a query compound encoded as an MHFP fingerprint. In order to compensate for approximation errors, $k_{c} \cdot k$ neighbors are searched for internally and their actual distance to the query molecule is computed using linear scan. $k_{c}$ is supplied as an optional parameter that defaults to $k_{c} = 10$. After this intermediate step, the top $k$ hits are then returned as the result of the LSH Forest query. Both scripts are available on GitHub (https://github.com/reymond-group/mhfp).

## Results and discussion

### Fingerprint design

The MinHash fingerprint (MHFP) described herein combines the concept of extended connectivity used for ECFP with MinHash as a hashing scheme to later enable LSH-based ANN searches. As a first step, we enumerate all circular substructures around each atom in a molecule and write these out as SMILES [6]. This operation yields $O\left( {n\left( {r + 1} \right)} \right)$ SMILES strings for a molecule with a heavy atom count (HAC) of $n$ and a maximum radius $r$. As for either small radii $r$ or macrocycles the ring information of a molecule is lost, we also extract the SMILES string for each ring of the symmetrized smallest set of smallest rings in the molecule. We then filter the SMILES strings for duplicates and combine them to a set $S\left( A \right)$ representing the molecular shingling of the molecule $A$.

We denote the process described above as “shingling of a molecule” and the resulting set *S(A)* as “molecular shingling”. A molecular shingling differs from the w-shingling of a document, where a w-shingling consists of n-grams with $n = w$, in that it includes SMILES strings of different lengths, with the maximum length depending on the maximum radius $r$ and the size of the rings in the molecule. The number of hashed unique SMILES-encoded molecular subgraphs with radius $r$ grows according to Heaps’ law with lower $\beta$ than ECFP hashes with radius $r$ when processing 1.7 million compounds from ChEMBL24 (Additional file 1: Fig. S1) [40, 41]. Given the molecular shinglings $S\left( {M_{a} } \right)$ and $S\left( {M_{b} } \right)$ of two molecules $M_{a}$ and $M_{b}$, the Jaccard similarity coefficient of the molecules is calculated according to Eq. 1 (see “Methods” section).

As the MinHash scheme cannot be applied directly to strings, the SMILES in a molecular shingling are first hashed to a 32-bit unsigned integer using a function $f:\varOmega \to \left\{ {0, \ldots ,2^{32} - 1} \right\}$. There is a trade-off when choosing this relatively small 32-bit hash, as the number of collisions (two or more different strings being hashed to the same integer value) during hashing is inversely proportional to the length of the hash. To estimate the number of collisions, molecular shingles with $r = 2$ were extracted from 1.7 million ChEMBL24 compounds, yielding a total number of 197,604 unique SMILES. Applying Eq. 3 (see “Methods” section), the number of expected collisions yields $c\left( {k = 197,604,N = 2^{32} - 1} \right) = 4.546.$ Increasing the maximum radius to $r = 3$ results in an increase to 2022,448 unique SMILES and 476.098 expected collisions. The measured numbers of collisions when hashing molecular shinglings from ChEMBL24 were 3 and 481 for $r = 2$ and $r = 3$, respectively, proving Eq. 3 to be a good estimator for SMILES hashing collisions. Substituting the 32-bit (SHA-1) hash with a 64-bit (SHA-1) hash would lower the number of estimated collisions to 0. However, a 64-bit hash would have the numbers of most calculations during MinHash computation exceed 64 bits, potentially slowing the MinHash computation and further processing by a factor of 2 on current hardware. In addition, the space requirement of the MinHash would double as well. Thus, SMILES contained within molecular shinglings are hashed to a 32-bit (SHA-1) hash.

To transform the hashed molecular shingling into our final fingerprints, we finally apply MinHash according to Eq. 2 (see “Methods” section). In the present study we calculated MinHash fingerprints for hashed molecular shinglings with $r \in \left\{ {2, 3, 4} \right\}$ and $k \in \left\{ {128, 1024, 2048, 4096} \right\}$. We considered radii $r = 2$ (MHFP4), $r = 3$ (MHFP6), and $r = 4$ (MHFP8), resulting in 12 fingerprints with different level of structural encoding and compression (according to common notation, the numbers in the fingerprint names represent the maximum diameter rather than the maximum radius).

### Benchmarking study

To validate the SMILES-strings based approach as well as the chosen hash function, we used a platform to benchmark fingerprints for ligand-based virtual screening with Jaccard similarity as a metric [7]. The benchmark performs statistically valid comparisons of fingerprints using structural and activity data drawn from DUD, MUV, and ChEMBL [40, 42, 43]. The benchmark evaluates 7 metrics: The area under the receiver operating characteristic (ROC) curve (AUC), the enrichment factor (EF) for $\chi = 0.01$ and $\chi = 0.05$, the Boltzmann-enhanced discrimination of ROC (BEDROC) for $\alpha = 20$ and $\alpha = 100$, and the robust initial enhancement (RIE) for $\alpha = 20$ and $\alpha = 100$.

**Fig. 2:**
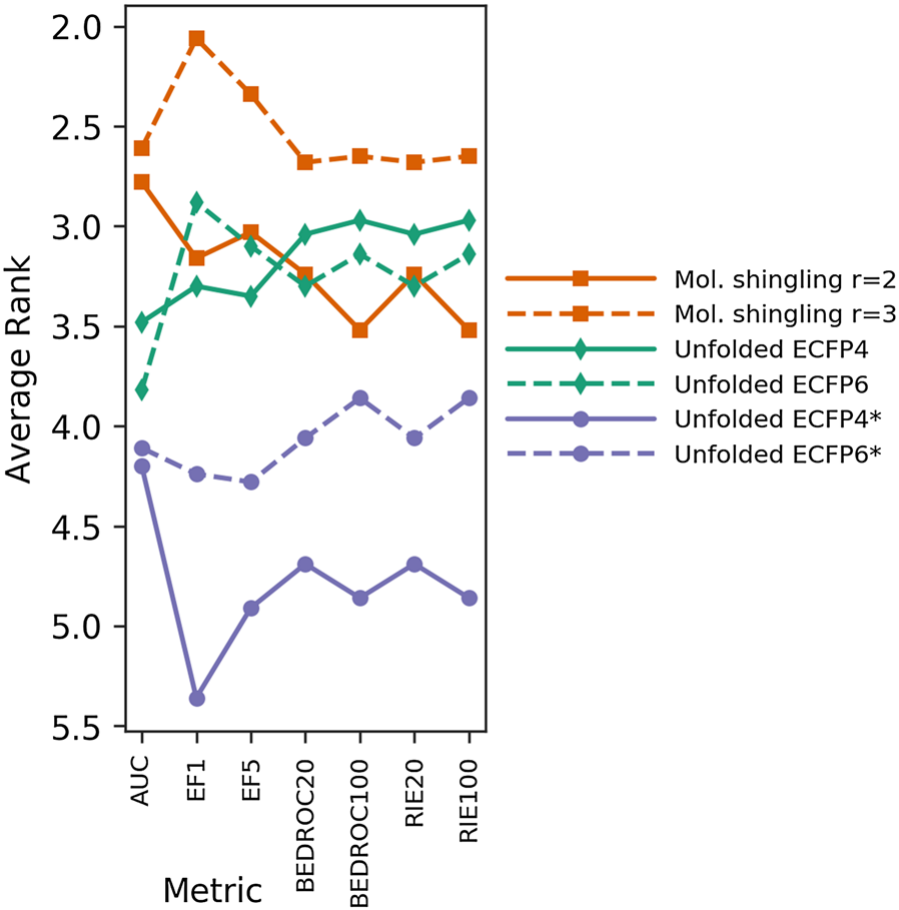
Results of benchmarking hashing methods across 88 benchmark targets. Hashed molecular shingling with $r = 2$ (orange, solid) and $r = 3$ (orange, dashed) are both ranked better than ECFP4/6 (green) and ECFP4/6* (purple) in AUC. However, only hashed molecular shingling with $r = 3$ was ranked better than all other fingerprints in every metric (AUC, EF1, EF5, BEDROC20, BEDROC100, RIE20, and RIE100). The control, a variant of ECFP, ECFP* (purple), considering only atomic numbers as invariants, performed significantly worse than both hashed molecular shingling and ECFP. Pairwise post hoc Friedman tests of the average rank were performed as part of the benchmark, resulting *p* values shown in Additional file 1: Fig. S5

First, we compared the hashed molecular shinglings to ECFP hashes before folding, as well as to ECFP*, a variant of ECFP considering only atomic numbers as invariants, all with $r = 2$ and $r = 3$. This comparison showed that the hashed molecular shingling method with a radius of $r = 3$ is superior to ECFP hashing, as it beats unfolded ECFP (with either radius $r = 2$ or $r = 3$) significantly in 2 out of 7 values (AUC, EF 5%) and with a *p* value above 0.05 in 5 out of 7 (EF 1%, BEDROC20, BEDROC100, RIE20 and RIE100) metrics (Fig. 2, Additional file 1: Fig. S5). ECFP* performed significantly worse with both $r = 2$ and $r = 3$ in all metrics compared to molecular shingling with $r = 3$.

**Fig. 3:**
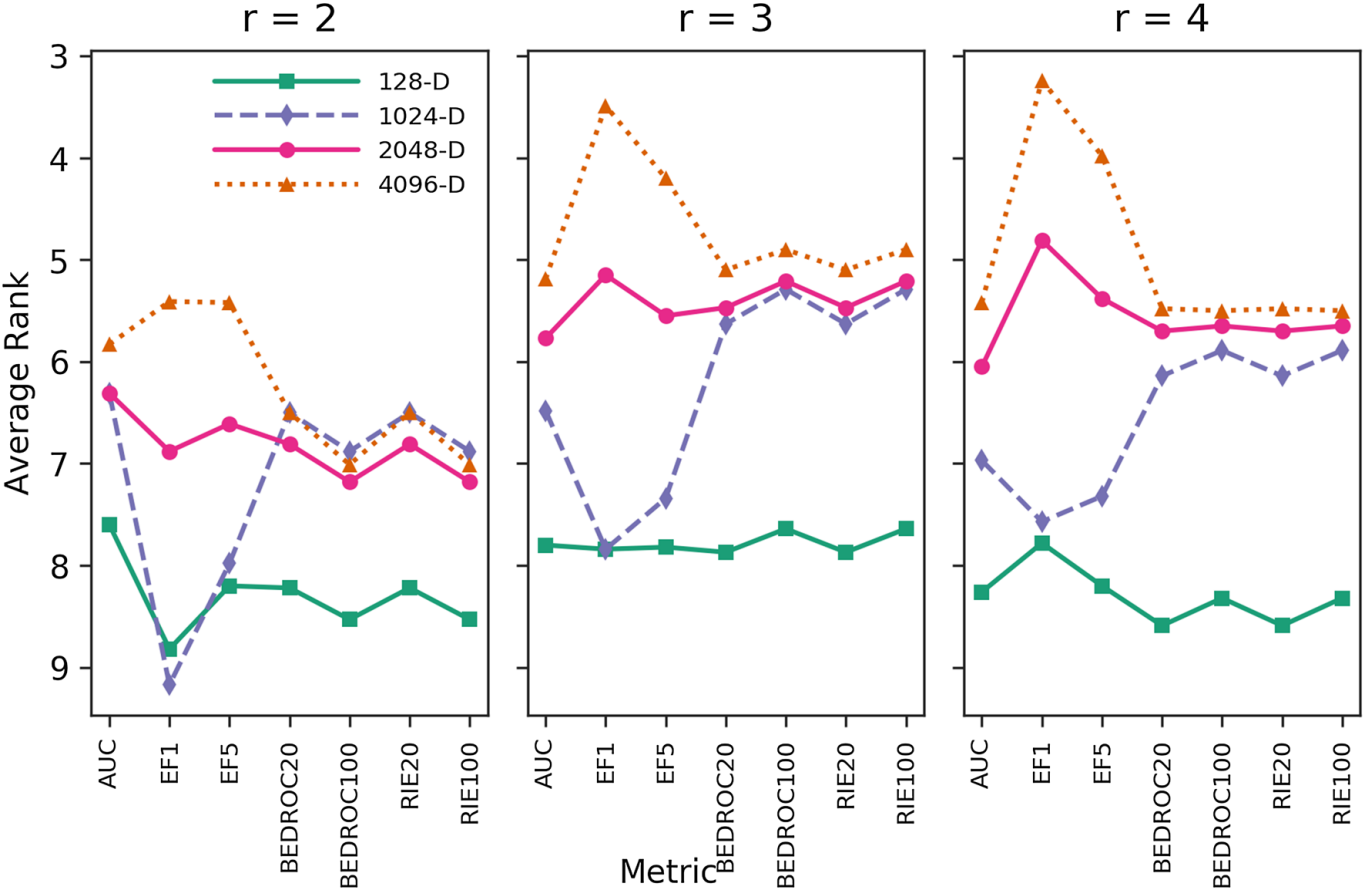
Average ranks of MHFP variants across 88 benchmark targets. Performance comparison of MHFP variants MHFP4/6/8 across dimensionalities 128-D, 1024-D, 2048-D, and 4096-D. While performance increases with an increase of the radius from $r = 2$ to $r = 3$, a further increase of the radius to $r = 4$ does not translate to further performance gains but a decrease, especially in BEDROC20, BEDROC100, RIE20 and RIE100 rankings. The benchmark used was a platform to benchmark fingerprints for ligand-based virtual screening with Jaccard similarity as a metric [7]

To establish whether results based on evaluating hashed molecular shinglings carry over to minhashed molecular shinglings, we then compared our 12 different MHFPs variants with each other. Comparing these different fingerprints in the benchmark confirmed that MHFP6 (MinHash applied to hashed molecular shinglings with $r = 3$) performed better than both MHFP4 ($r = 2$) and MHFP8 ($r = 4$) for medium (1024-D, 2048-D) to high dimensional (4096-D) variants (Fig. 3). The data further suggested that low dimensional variants such as 128-D perform better with $r = 2$. As MHFP8 failed to perform better than MHFP6, it was discounted from further experiments. MHFP4, while also performing worse than MHFP6, was kept for further experiments as a comparison to ECFP variants with $r = 2$.

Given the results of benchmarking unfolded ECFP hashes and hashed molecular shinglings (Fig. 2), as well as the results of benchmarking different MHFP radii (Fig. 3), we finally selected the following fingerprints for a detailed comparison aimed at identifying the best fingerprint: (1) Folded ECFP4 and ECFP6; (2) MinHash molecular shinglings with radii 2 and 3, henceforth denoted MHFP4 and MHFP6; (3) MinHash ECFP4 and ECFP6, henceforth denoted MHECFP4 and MHECFP6, respectively, used here to control for the performance of encoding SMILES (MHFP) as opposed to hashes of invariants (ECFP) by applying the minhashing scheme to unfolded ECFP values (Fig. 1). For each fingerprint four different dimensionalities were evaluated.

**Fig. 4:**
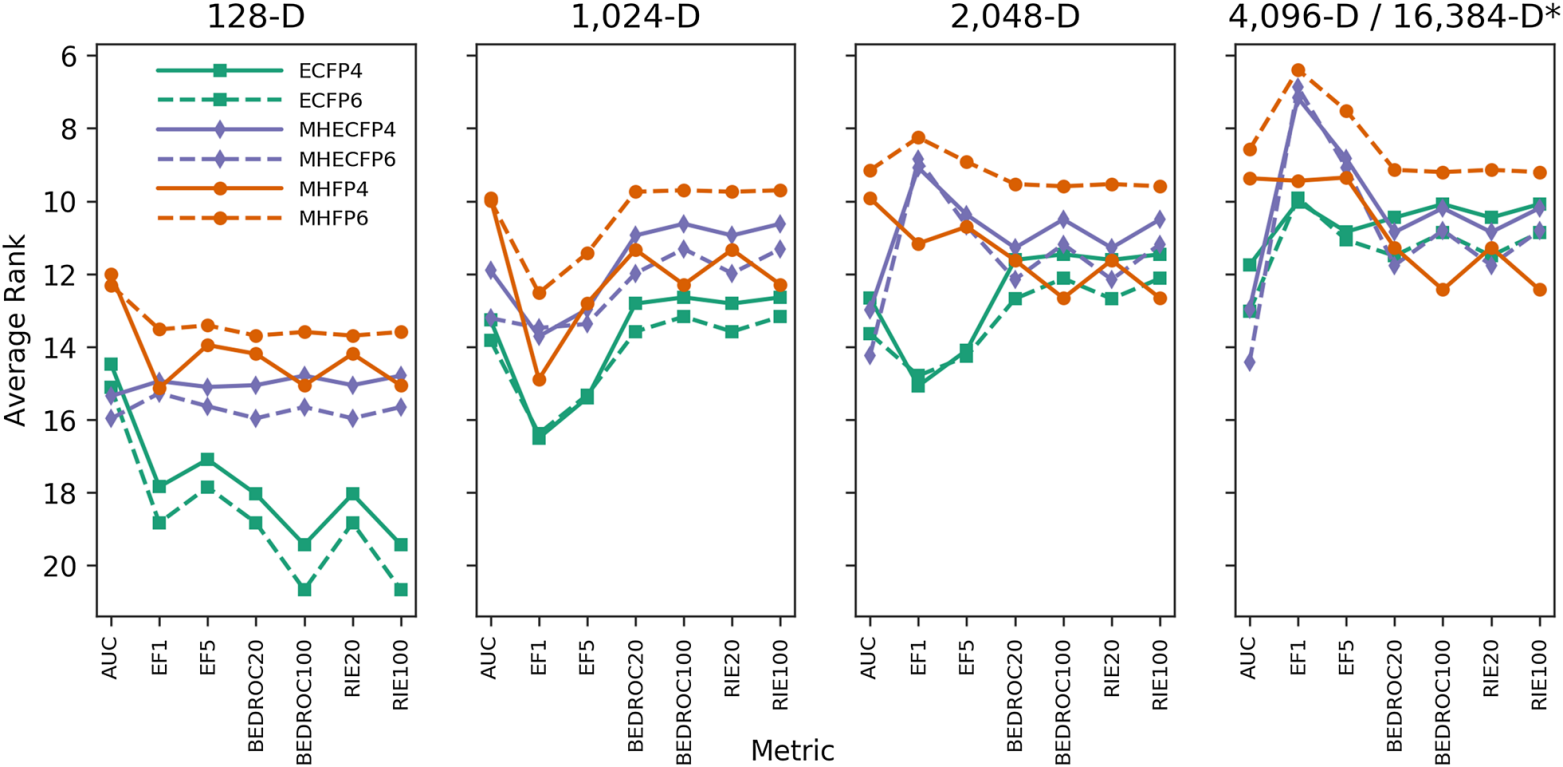
Average ranks of ECFP4/6, MHECFP4/6 and MHFP4/6 across 88 benchmark targets. The benchmark was run for a total of 24 fingerprint variants. MHFP6 generally outperforms MHFP4, while ECFP4 and MHECFP4 are always ranked equal or better than ECFP6 and MHECFP6, respectively. MHFP6 matches or outperforms ECFP4/6 and MHECFP4/6 in virtually all metrics across benchmarked dimensionalities (pairwise post hoc Friedman tests of the average rank were performed as part of the benchmark, resulting *p* values in Additional file 1: Fig. S6). (*) The 4096-D variants of MHECFP4/6 and MHFP4/6 were compared to the 16,384-D variant of ECFP4/6 as this is the highest reported dimensionality applied with ECFP

An average rank comparison according to the benchmark is shown in Fig. 4. Comparing the average ranking of the fingerprints as a function of the chosen radius, both ECFP4 and MHECFP4 perform marginally better than their respective counterparts, ECFP6 and MHECFP6, in the vast majority of cases. In contrast, MHFP6 generally performs better than MHFP4. This result confirms the observations from Fig. 2 where hashed molecular shinglings performed better with $r = 3$ than with $r = 2$, while the ECFP4 hashes outperformed ECFP6 hashes. With the exception of the 128-D variant, MHFP4/6 exhibit strictly better performance in AUC compared to both MHECFP4/6 and ECFP4/6, while both MHFP4/6 and MHECFP4/6 perform better than ECFP4/6 in early recognition metrics EF1 and EF5, suggesting that the AUC performance gains are a result of the molecular shingling approach, while the gains in early recognition can be attributed to minhashing. Note that MHFP6 (both 2048-D and 4096-D) did not perform significantly worse than path-based methods (TT and AP, [7]) in AUC, while performing generally significantly better in other metrics, which is in contrast to ECFP fingerprints, which perform worse in AUC benchmarks than path based fingerprints (Additional file 1: Fig. S4).

**Fig. 5:**
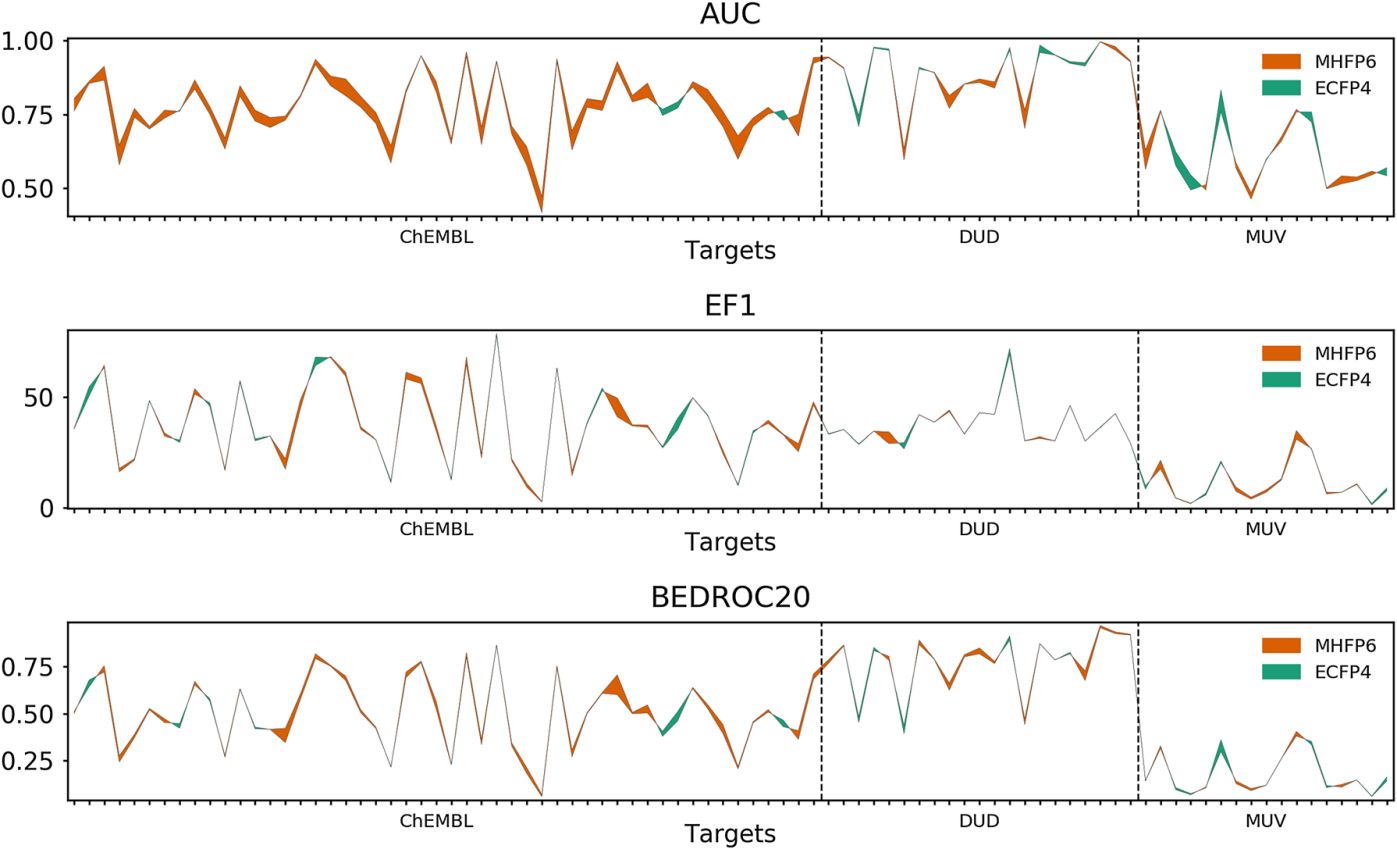
Performance comparison between MHFP6 2048-D and ECFP4 2048-D. Colors highlighting the difference in the AUC, EF1 and BEDROC20 values for 88 targets between MHFP6 2048-D (orange) and ECFP4 2048-D (green). MHFP6 significantly outperforms MHECFP4 in the AUC and EF1 metrics (see pairwise post hoc Friedman tests of the average rank results in Additional file 1: Fig. S6a). Comparisons to EF5, BEDROC100, RIE20 and RIE100 can be found in Additional file 1: Fig. S2, comparisons of all metrics between MHFP6 and MHECFP4 (both 2048-D) in Additional file 1: Fig. S3

The above comparisons established that MHFP6 provided the best overall performance across all fingerprints considered, with the 2048-D offering a good compromise between performance and size. In detail, 2048-D MHFP6 significantly outperformed 2048-D ECFP4 in AUC, EF1 and EF5, while performing non-significantly better in BEDROC20, BEDROC100, RIE20 and RIE100. In fact, 2048-D MHFP6 was comparable to 16,384-D ECFP4, although it still performed better in terms of BEDROC20 and RIE20. 2048-D MHFP6 also performed significantly better in AUC than 2048-D MHECFP4 while non-significantly better in EF1, EF5, BEDROC100 and RIE100 and worse in BEDROC20 and BEDROC100. While 2048-D MHFP6 ranked significantly worse than 4096-D MHECFP4 in AUC, 4096-D MHFP6 significantly outranked 4096-D MHECFP4 in AUC (Additional file 1: Fig. S6). Further analysis of the data suggested that gains by MHFP6 over ECFP4 was largely due to better performance on benchmark targets selected from ChEMBL24, while performing approximately equal on DUD and MUV data (Fig. 5, see full target-level performance comparisons between 2048-D MHFP6 and 2048-D ECFP4 and MHECFP4 in Additional file 1: Figs. S2 and S3, respectively).

**Fig. 6:**
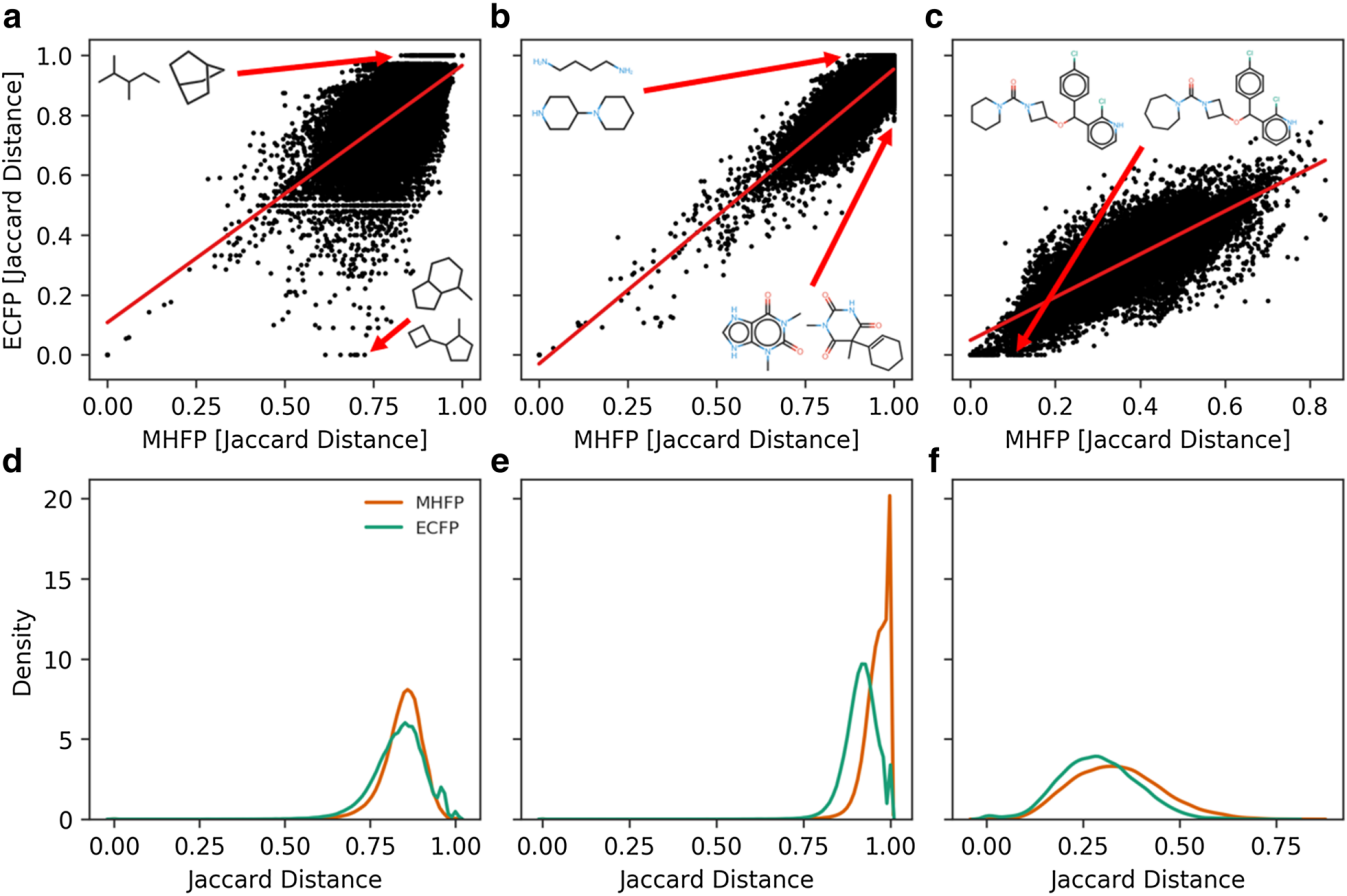
Comparing measured distances between MHFP6 and ECFP4 (2048-D) in different data sets. The distances in all data sets show moderate to strong linear correlation of r = 0.659, r = 0.792, and r = 0.829 for GDB-13 hydrocarbons, Drugbank, and MMP respectively. **a**, **d** While the distribution of measured distances is similar for MHFP6 and ECFP4 for the GDB-13 subset, ECFP4 seems to measure a distance of 0.0 between clearly different molecules. Gaps appear in measured ECFP4 distances, resulting in a multimodal distribution. **b**, **e** The distances measured in Drugbank show a strong correlation. Both fingerprints seem to measure a distance of 1.0 in molecules where a finer grained distance measure could proof beneficial. **c**, **f** The MMP data set exposes the inability of ECFP4 to distinguish between highly similar molecules that differ only in the size of one ring whereas MHFP6 seems to express higher resolution for distance measurements between very similar compounds

To further compare MHFP6 and ECFP4, we explored the respective Jaccard distance measurements between molecules within three sets: (1) A subset of hydrocarbons extracted from GDB-13 ($n = 3,824$), (2) Drugbank ($n = 9,300$), and (3) a matched molecular pairs (MMP) set ($n = 240,322$) [44–46]. For the hydrocarbon and Drugbank sets, 50 compounds were randomly selected from each, and their Jaccard distance to all the compounds in their respective set was computed. In the MMP set, the Jaccard distance between each pair was computed. While the distances in all data sets show moderate to strong linear correlation ($r = 0.659$, $r = 0.792$, and $r = 0.829$ for GDB-13 hydrocarbons, Drugbank, and MMP respectively), we observed interesting differences. While the distribution of measured distances is similar for MHFP6 and ECFP4 for the GDB-13 subset, ECFP4 seems to measure a distance of 0.0 between clearly different molecules (Fig. 6a, d). In addition, gaps appear in measured ECFP4 distances, resulting in a multimodal distribution—an effect that cannot be fully attributed to the folding operation of ECFP4, as MHECFP4 shows a similar pattern (Additional file 1: Fig. S8a, d). The distances measured in Drugbank show a strong correlation, however, both fingerprints seem to measure a distance of 1.0 in molecules where a finer grained distance measure could prove beneficial (Fig. 6b, e). The MMP data set exposes the inability of ECFP4 to distinguish between highly similar molecules that differ only in the size of one ring compared to MHFP6, which seems to express higher resolution for distance measurements between very similar compounds.

**Fig. 7:**
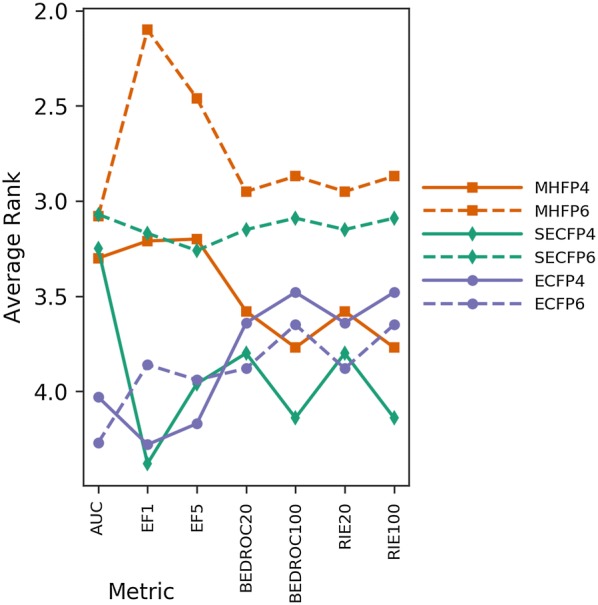
Average ranks of 2048-D ECFP4/6, MHFP4/6 and SECFP4/6 across 88 benchmark targets. The SMILES-based circular substructure hashing scheme applied by MHFP was folded using ECFP’s module $n$ method. This fingerprint variant was denoted SECFP. While SECFP4/6 were outperformed by MHFP4/6 respectively, SECFP6 performed significantly better than both ECFP4/6. These results suggest that SECFP6 can be readily used as a drop-in replacement for ECFP with beneficial results. By performing significantly worse compared to MHFP6, acting as a control, SECFP6 further validates the minhashing approach as compared to folding

As MHFP6 significantly outperformed both MHECFP4 and ECFP4 (Figs. 4, 5, and Additional file 1: S6), a fingerprint variant on MHFP’s SMILES-based circular substructure hashing scheme, folded by the same modulo $n$ operation that is used by ECFP, was compared to both minhashed MHFP and folded ECFP with $r \in \left\{ {2, 3} \right\}$ and $D = 2048$ (Fig. 7). We denoted this variant SECFP (SMILES extended connectivity fingerprint). While SECFP4/6 were outperformed by MHFP4/6 respectively, SECFP6 performed significantly better than both ECFP4/6 (Additional file 1: Fig. S9). These results suggest that SECFP6 can be readily used as a drop-in replacement for ECFP4 with beneficial results. By performing significantly worse compared to MHFP6, acting as a control, SECFP6 further validates the minhashing approach as compared to folding (Additional file 1: Fig. S10). However, as the minhashed MHFP is based on a sparse representation of the $2^{32}$-dimensional binary hash space with a fixed number ($D$) of set bits, search optimization algorithms assuming $D$-dimensional binary vectors such as BitBound cannot be applied to it.

### Approximate k-nearest neighbor (ANN) searches

In the context of big data, the key advantage of our MHFP over ECFP consists in the implementation of MinHash (Fig. 7), which enables the use of the LSH Forest algorithm to perform ANN searching in the sparse, $2^{32}$-dimensional hash space. As a comparison, ECFP hashes are folded into binary arrays, indexed and searched using the ANN algorithm Annoy [33, 36]. Annoy is used by the R package eiR for accelerated structure similarity searching of very large small molecule data sets [26]. To establish whether the performance of LSH Forest can be compared to that of state-of-the-art ANN algorithms when indexing chemical fingerprints, we compared 2048-D MHFP6 fingerprints and 2048-D ECFP4 fingerprints indexed by LSH Forest and Annoy, respectively. A benchmark based on all compounds found in ChEMBL24 ($n = 1,712,978$) was set up. From ChEMBL24, 20 compounds were randomly selected as query compounds. Next, for each of the 20 query compounds, the Jaccard distances to all compounds from ChEMBL24 were calculated using brute-force linear scan, resulting in 20 sorted lists. These steps were performed for MHFP6, and ECFP4 based Jaccard distances. Finally, the recovery rates of $k$-nearest neighbors for $k \in \left\{ {5, 10, 50, 100} \right\}$ of approximate $k$-nearest neighbor algorithms (LSH Forest for MHFP6 and Annoy for ECFP4) were calculated and the respective query times measured. For each value of $k$, the benchmark was repeated over parameter $k_{c} \in \left\{ {1, 10, 20, \ldots , 90, 100} \right\}$.

**Fig. 8:**
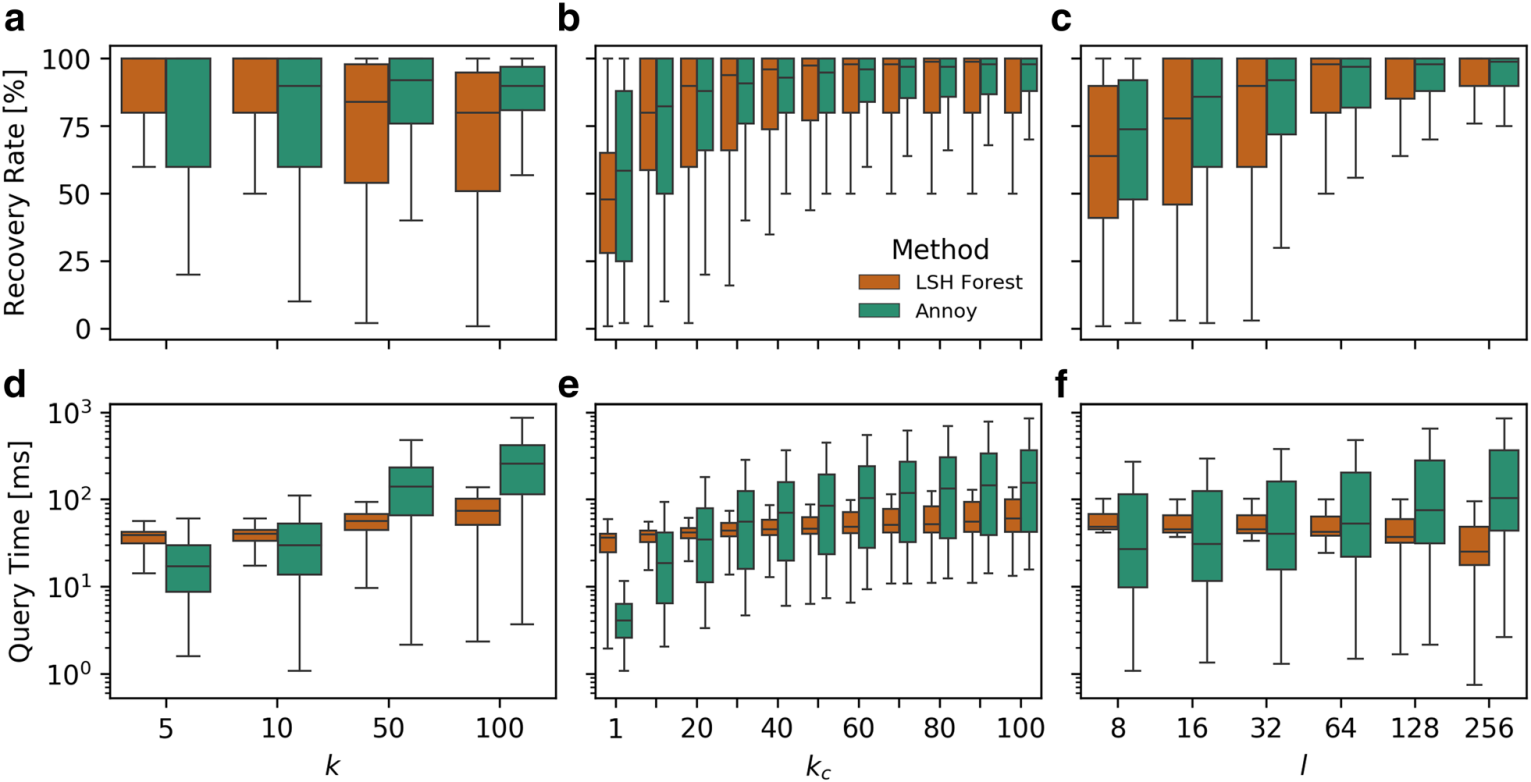
ChEMBL ($n = 1.7M$) k-nearest neighbor searches performance of 2048-D MHFP6 indexed using LSH Forest and 2048-D ECFP4 indexed using Annoy Recovery rates for both implementations depend on parameters $k$, $k_{c}$, and $l$. **a** While LSH Forest performs better for $k = 5$ and $k = 10$ nearest neighbors, Annoy surpasses LSH Forest for $k = 50$ and $k = 100$. **b** By increasing the number of nearest neighbors by a factor of $k_{c}$, the performance of both ANN neighbor methods can be greatly improved. While LSH Forest (orange) shows worse performance compared to Annoy (green) for $k_{c} < 20$, it surpasses Annoy for higher values. **c** Increasing the number of trees $l$ increases the recovery rate for both methods at the expense of main memory. Annoy performs slightly better for $l = 8, \ldots ,128$, performance of LSH Forest increases at a greater rate, overtaking Annoy at $l = 256$. **d**, **e** Increasing values of parameters $k_{c}$ and $k$ affects query times of Annoy negatively. While the average query time for LSH Forest remains below 100 ms for $k = 50$ and $k = 100$, Annoys average query time increases to above 100 and 200 ms respectively. **f** As the number of prefix trees, and thus the recovery rate, in LSH Forest increases, the query time decreases. On the other hand, an increase in Annoy trees, with a beneficial effect on recovery rate, also increases the query time. For subplots **a**, **d**; **b**, **e**; and **c**, **f**; the data has been aggregated over all measured values for $k_{c}$, $l$; $k$, $l$; and $k_{c}$, $k$; respectively

LSH Forest and Annoy were each benchmarked with $l \in \left\{ {8, 16, 32, 64, 128, 256} \right\}$ prefix and Annoy trees, respectively. While LSH Forest performs better for $k = 5$ and $k = 10$ nearest neighbors, Annoy surpasses LSH Forest for $k = 50$ and $k = 100$ (Fig. 8a). By increasing the number of nearest neighbors by a factor of $k_{c}$, the performance of both ANN neighbor methods can be greatly improved. LSH Forest (orange) shows worse performance compared to Annoy (green) for $k_{c} < 20$, however, for $k_{c} \ge 20$ it surpasses Annoy (Fig. 8b). As LSH Forest and Annoy both construct multiple trees (prefix and binary trees respectively) in order to approximate optimal nearest neighbor search, increasing the number of trees $l$ increases the recovery rate for both methods at the expense of main memory. Annoy performs slightly better for $l = \left\{ {8, \ldots ,128} \right\}$, however, performance of LSH Forest increases at a greater rate, overtaking Annoy at the final value of $l = 256$ (Fig. 8c).

Increasing values of parameters $k_{c}$ and $k$ affects query times of Annoy negatively, while the average query time for LSH Forest only shows a small increase and remains below 100 ms for $k = 50$ and $k = 100$, Annoys average query time increases to above 100 and 200 ms respectively (Fig. 8d, e). The comparatively steep increase in query time for Annoy with $k_{c} > 1$ is caused by cosine similarity computations, which are more resource demanding than Jaccard distance computations. A major difference between the two methods is the effect of parameter *l* on query time. As the number of prefix trees *l*, and thus the recovery rate, in LSH Forest increases, the query time decreases. On the other hand, an increase in Annoy trees, while having a beneficial effect on recovery rate, has a negative effect on query time (Fig. 8f).

The combination of MHFP and LSH Forest allows for fast and accurate searching in sparse, high-dimensional binary chemical spaces. Its performance is comparable to methods such as Annoy which rely on the folding of fingerprint vectors, although the presented implementation is limited in terms of speed and scope of data set size due to in-memory processing and Python.

## Conclusion

MHFP6 is a new fingerprint based on the circular nature of ECFP combined with methods from natural language processing and data mining. The data presented here and the earlier benchmark study [7] demonstrate that MHFP6 performs better than any currently used fingerprint in a ligand-based virtual screening. Furthermore, MHFP6 enables approximate $k$-nearest neighbor searches in sparse and high-dimensional binary chemical spaces without folding through the direct application of ANN algorithms such as LSH Forest, thereby successfully removing the curse of dimensionality while preserving locality. In addition to improving $k$-nearest neighbor search speed by two orders of magnitude, LSH Forest, in combination with MHFP6, also has the potential to significantly increase search accuracy compared to other methods for ANN. The remarkable performance of MHFP6 makes the new fingerprint a highly recommended alternative to ECFP4 for virtual screening experiments as well as for querying and analyzing large chemical databases. Furthermore, the input agnostic MinHash encoding scheme facilitates the creation of use-case based variants of the fingerprint through the inclusion of additional chemically relevant features. The source code for MHFP6 is available on GitHub (https://github.com/reymond-group/mhfp).

## Additional file

**Additional file 1.** **Figure S1.** Number of ECFP and substructure SMILES hashes extracted from ChEMBL. **Figure S2.** Performance comparison between MHFP6 2048-D and ECFP4 2048-D. **Figure S3.** Performance comparison between MHFP6 2048-D and MHECFP4 2048-D. **Figure S4.** Average ranks of (L)ECFP4, (L)MHFP6, and path-based methods across 88 benchmark targets. **Figure S5.** Results of benchmarking hashing methods across 88 benchmark targets. **Figure S6.** Pairwise post-hoc Friedman tests of the average rank of MHFP4/6. **Figure S7.** Pairwise post-hoc Friedman tests of the average rank of MHFP4/6 (path-based methods). **Figure S8.** Comparing measured distances between MHFP6 and MHECFP4 (2048-D) in different data sets. **Figure S9.** Pairwise post-hoc Friedman tests of the average rank of SECFP4/6. **Figure S10.** Pairwise post-hoc Friedman tests of the average rank of MHFP4/6.

## Abbreviations

ANN: approximate nearest neighbor
AP: atom pair fingerprint
AUC: area under the curve
BEDROC: Boltzmann-enhanced discrimination of the receiver operating characteristic
DUD: directory of useful decoys
DUD-E: directory of useful decoys enhanced
ECFP: extended connectivity fingerprint
EF: enrichment factor
GDB: generated database
HAC: heavy atom count
LSH: locality sensitive hashing
MHECFP: MinHash extended connectivity fingerprint
MHFP: MinHash fingerprint
MQN: molecular quantum numbers
MUV: maximum unbiased validation data sets
RIE: robust initial enhancement
ROC: receiver operating characteristic
SECFP: SMILES extended connectivity fingerprint
SMIfp: SMILES fingerprint
SMILES: simplified molecular-input line-entry system
TT: topological torsion fingerprint
ZINC: ZINC is not commercial
