# Supplementary material for: A probabilistic molecular fingerprint for big data settings
Source: J Cheminform. 2018 Dec 18;10:66. doi: 10.1186/s13321-018-0321-8 (PMC6755601; doi:10.1186/s13321-018-0321-8)
Supplement: Supplementary file 1 — Additional file 1. Figure S1. Number of ECFP and substructure SMILES hashes extracted from ChEMBL. Figure S2. Performance comparison between MHFP6 2048-D and ECFP4 2048-D. Figure S3. Performance comparison between MHFP6 2048-D and MHECFP4 2048-D. Figure S4. Average ranks of (L)ECFP4, (L)MHFP6, and path-based methods across 88 benchmark targets. Figure S5. Results of benchmarking hashing methods across 88 benchmark targets. Figure S6. Pairwise post-hoc Friedman tests of the average rank of MHFP4/6. Figure S7. Pairwise post-hoc Friedman tests of the average rank of MHFP4/6 (path-based methods). Figure S8. Comparing measured distances between MHFP6 and MHECFP4 (2048-D) in different data sets. Figure S9. Pairwise post-hoc Friedman tests of the average rank of SECFP4/6. Figure S10. Pairwise post-hoc Friedman tests of the average rank of MHFP4/6. [file 13321_2018_321_MOESM1_ESM.docx]

**Supplementary Information**


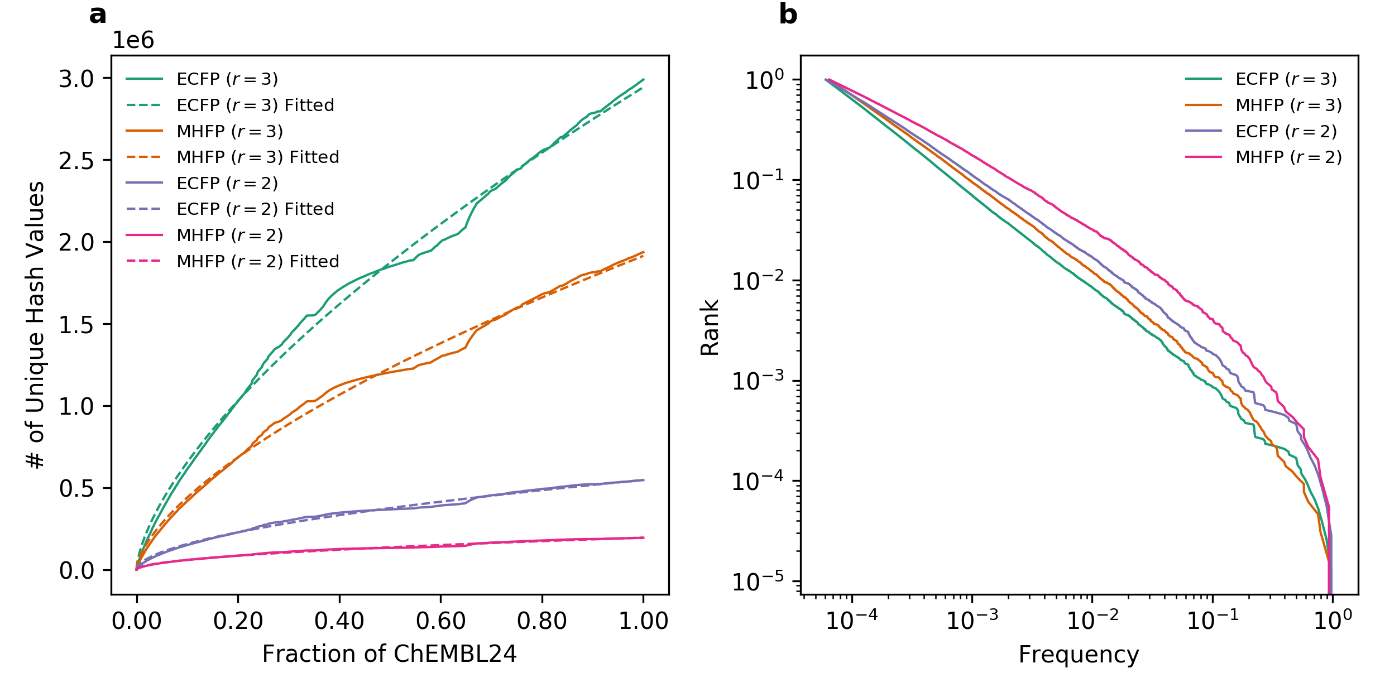


**Figure S1 Number of ECFP and substructure SMILES hashes extracted from ChEMBL**. (a) For both radii $r=2$ and $r=3$, the number of unique substructure SMILES hashes extracted from ChEMBL (pink and orange) is lower than the number of unique ECFP hashes (purple and green). However, both approaches exhibit a similar pattern as can be seen by their deviations from the fitted curve between fractions 0.2 and 0.7. All four variants follow Heap’s law with $\beta=0.652$, $0.638$, $0.544$, and $0.515$ for ECFP6, MHFP6, ECFP4, and MHFP4 respectively. (b) The associated Zipf plot shows the expected rank/frequency distributions [41].


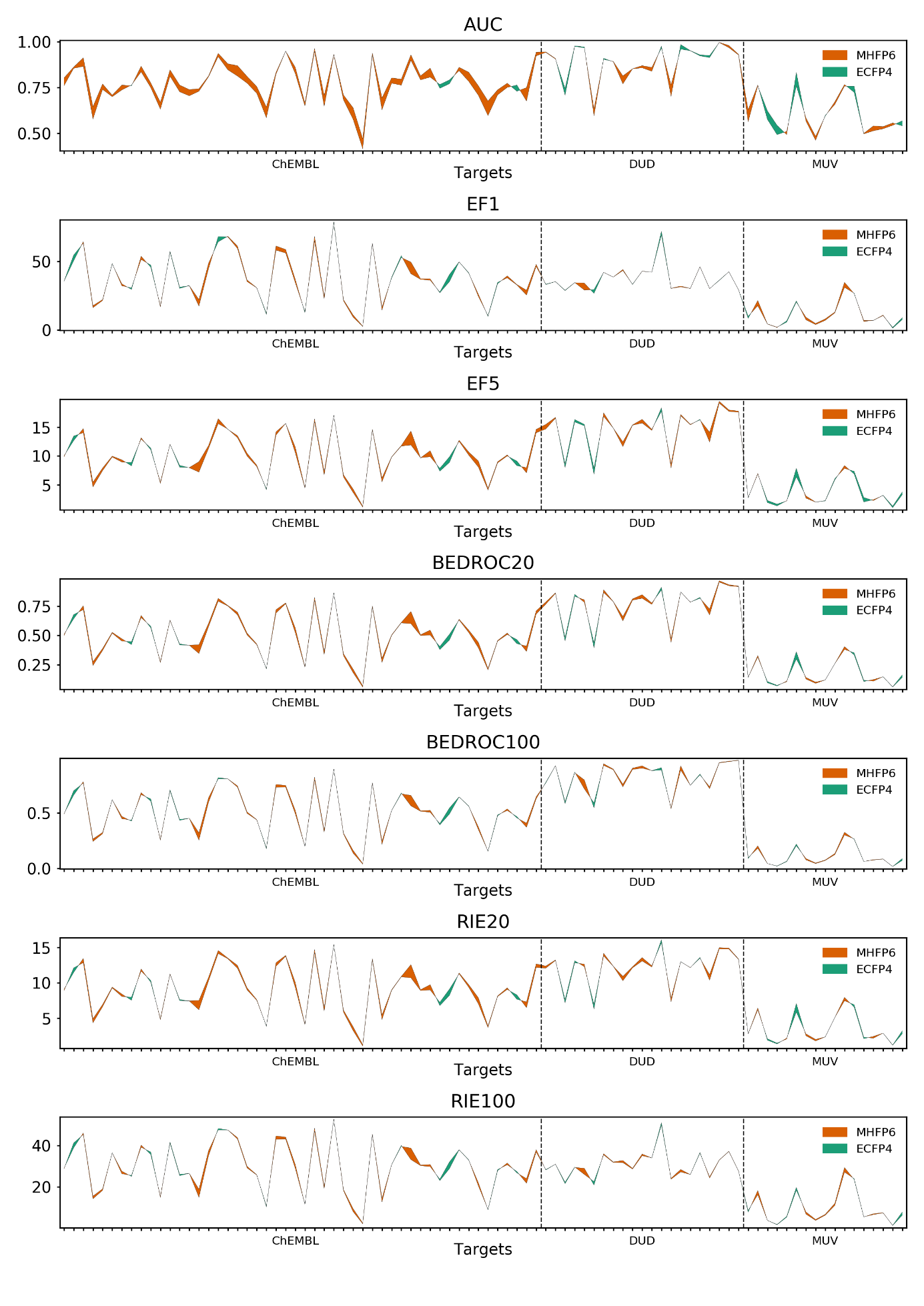


**Figure S2 Performance comparison between MHFP6 2,048-D and ECFP4 2,048-D**. Colors highlighting the difference in the AUC, EF1, EF5, BEDROC20, BEDROC100, RIE20 and RIE100 values for 88 targets between MHFP6 2,048-D (orange) and ECFP4 2,048-D (green). MHFP6 significantly outperforms ECFP4 in the AUC, EF1 and EF5 metrics (see pairwise post-hoc Friedman tests of the average rank results in Figure S6a).


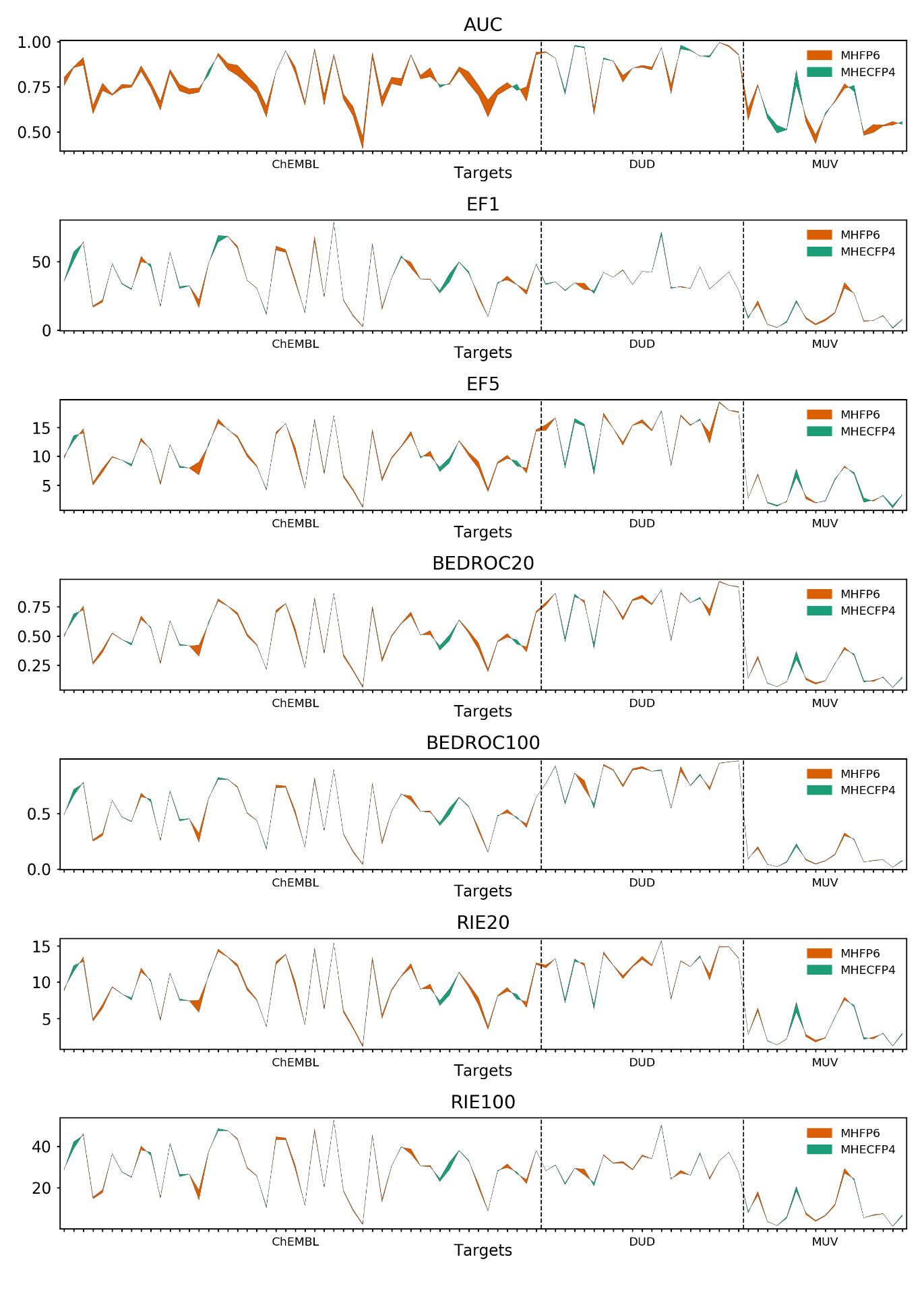


**Figure S3 Performance comparison between MHFP6 2,048-D and MHECFP4 2,048-D**. Colors highlighting the difference in the AUC, EF1, EF5, BEDROC20, BEDROC100, RIE20 and RIE100 values for 88 targets between MHFP6 2,048-D (orange) and MHECFP4 2,048-D (green). MHFP6 significantly outperforms MHECFP4 in the AUC, EF1 and EF5 metrics (see pairwise post-hoc Friedman tests of the average rank results in Figure S6a).


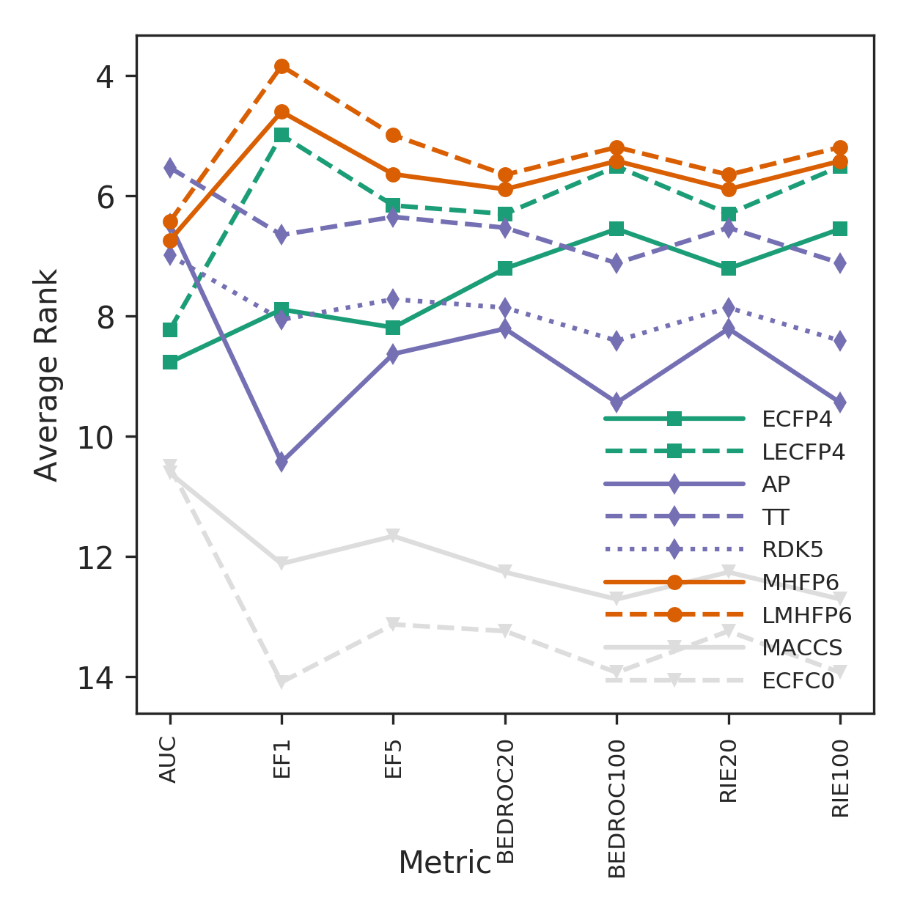


**Figure S4 Average ranks of (L)ECFP4, (L)MHFP6, and path-based methods across 88 benchmark targets**. Comparisons to path-based methods AP (Atom Pair fingerprint), TT (Topological Torsion fingerprint) and RDK5 (RDKit implementation of the Daylight fingerprint) show that the AUC performance of (L)MHFP6 is not significantly different from that of path-based methods but significantly better than that of (L)ECFP4 (Figure S7). However, (L)MHFP6 outperforms path-based methods in other metrics. ECFP4 and LECFP4 are 1,024-D and 16,384-D, respectively. MHFP4 and LMHFP4 correspond to 2,048-D and 4,096-D, respectively. MACCS and ECFC0 were used as a baseline.


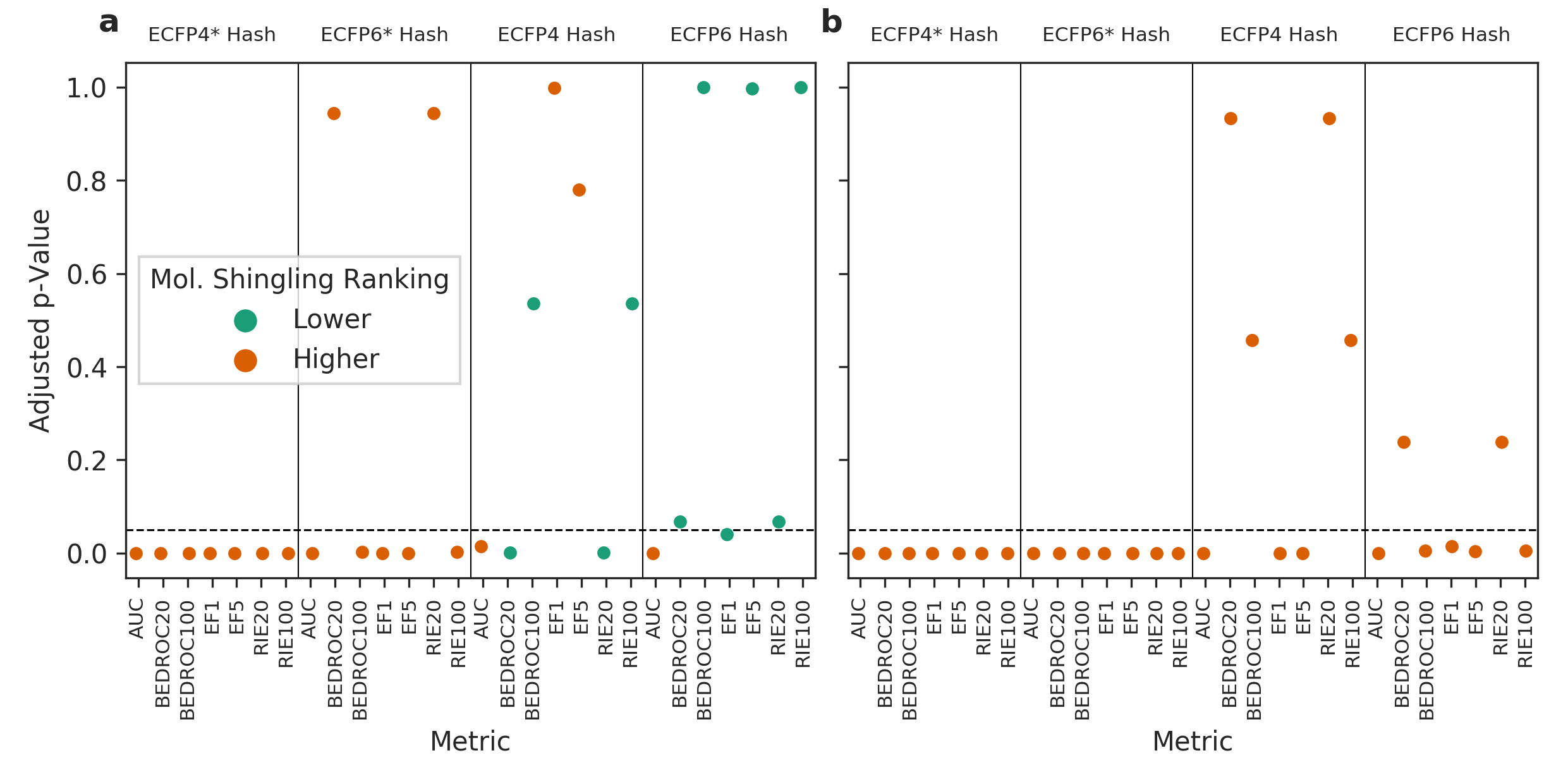


**Figure S5 Results of benchmarking hashing methods across 88 benchmark targets.** Results from benchmarking hashing approaches without further dimensionality reduction [7]. (a) Comparison of hashed molecular shinglings with $r=2$ with ECFP4/6* and ECFP4/6 hashes. (b) Comparison of hashed molecular shinglings with $r=3$ with ECFP4/6* and ECFP4/6. As a control, variants of ECFP4/6, ECFP4/6*, considering only atomic numbers as invariants was benchmarked. Green and orange colors indicate molecular shingling hashes being ranked lower and higher than ECFP hashes, respectively. Data points below the dashed line are $0.05$.


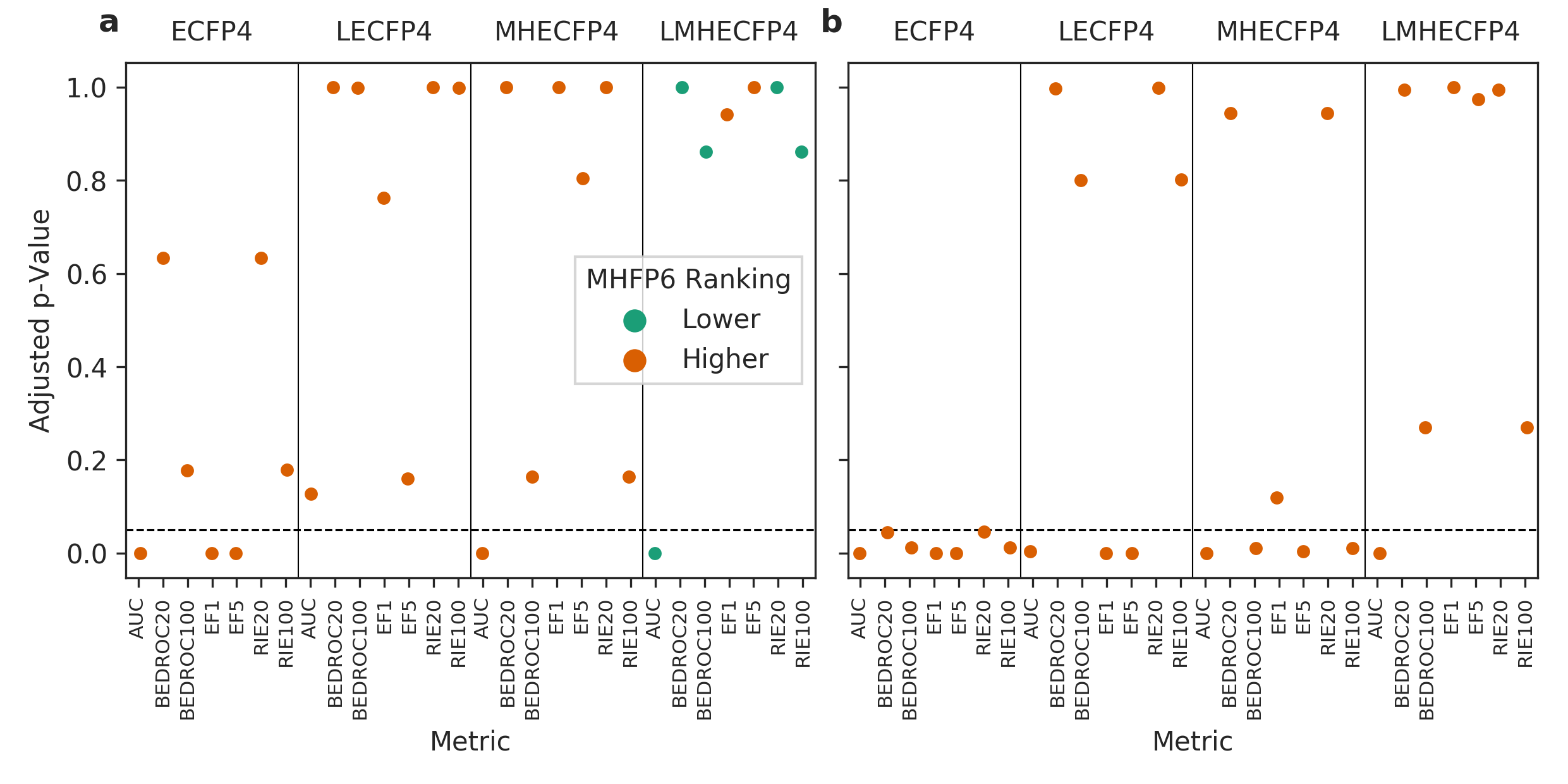


**Figure S6 Pairwise post-hoc Friedman tests of the average rank of MHFP4/6**. Statistical tests were run as part of the benchmark and visualized for easier comprehension. (a) Relative ranking and p-values of 2,048-D MHFP6 compared to ECFP4 (2,048-D), LECFP4 (16,384-D), MHECFP4 (2,048-D), and LMHECFP4 (4,096-D). (b) Relative ranking and p-values of 4,096-D MHFP6 compared to ECFP4 (2,048-D), LECFP4 (16,384-D), MHECFP4 (2,048-D), and LMHECFP4 (4,096-D). Orange color corresponds to MHFP6 being ranked higher than the other fingerprint, while green color indicates a lower ranking. P-values below 0.05 (dashed horizontal line) indicate significance.


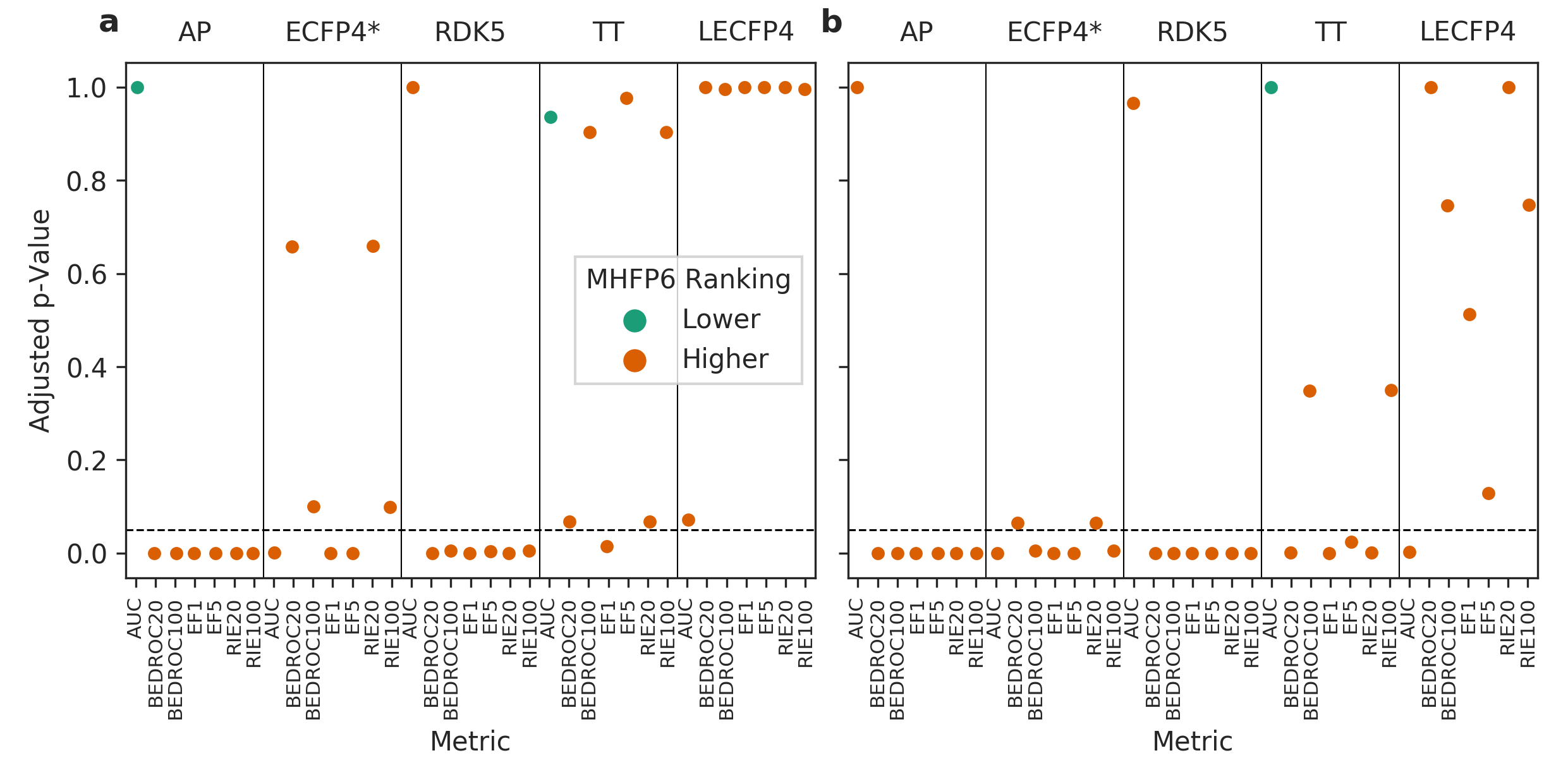


**Figure S7 Pairwise post-hoc Friedman tests of the average rank of MHFP4/6 (path-based methods)**. Statistical tests were run as part of the benchmark and visualized for easier comprehension. (a) Relative ranking and p-values of 2,048-D MHFP6 compared to AP (Atom Pair fingerprint), ECFP4 (1,024-D), RDK5 (RDKit implementation of the Daylight fingerprint) TT (Topological Torsion fingerprint), and LECFP4 (16,384-D). (b) Relative ranking and p-values of 4,096-D MHFP6 compared to AP (Atom Pair fingerprint), ECFP4 (1,024-D), RDK5 (RDKit implementation of the Daylight fingerprint) TT (Topological Torsion fingerprint), and LECFP4 (16,384-D). Orange color corresponds to MHFP6 being ranked higher than the other fingerprint, while green color indicates a lower ranking. P-values below 0.05 (dashed horizontal line) indicate significance.


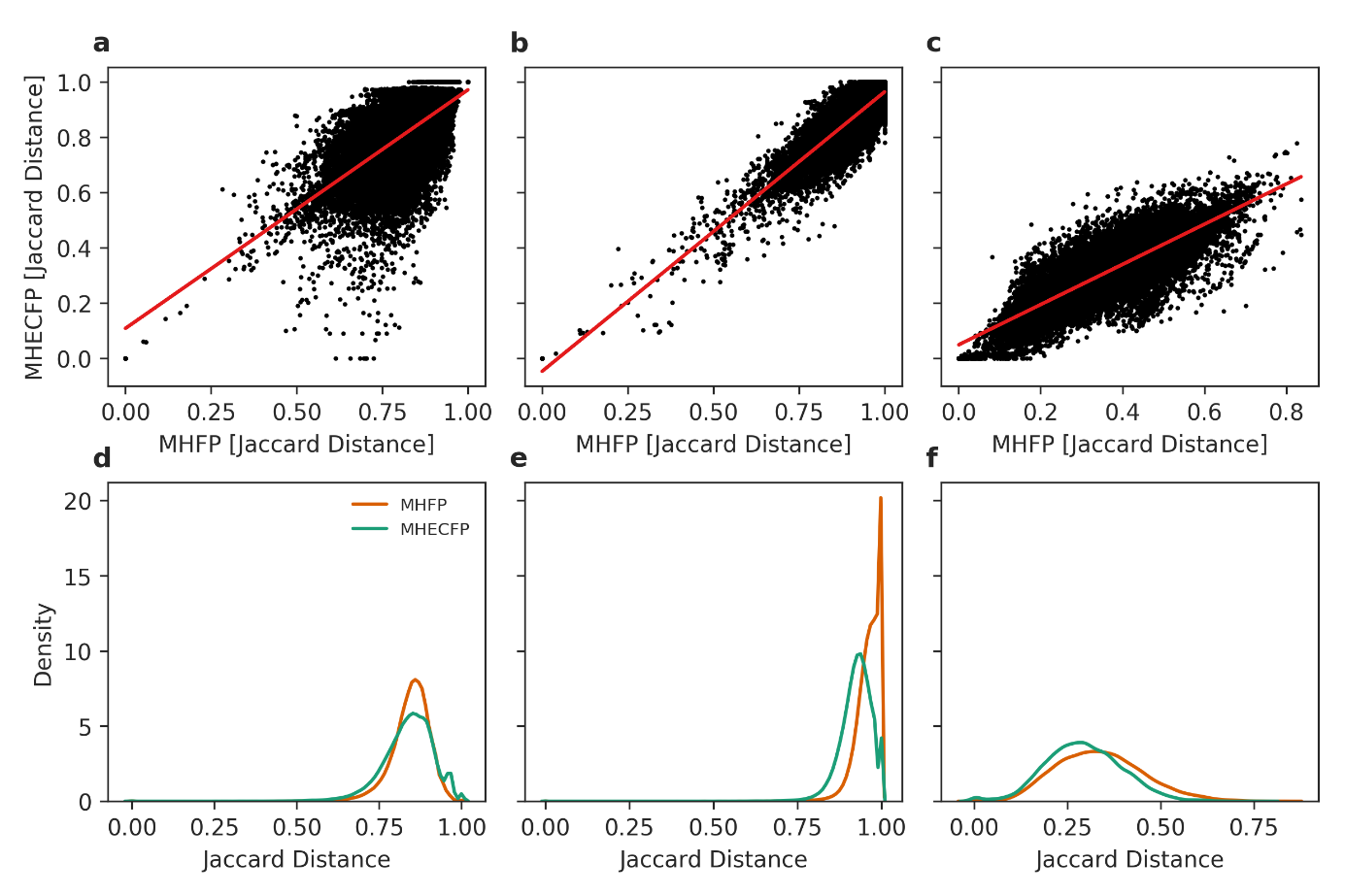


**Figure S8 Comparing measured distances between MHFP6 and MHECFP4 (2,048-D) in different data sets.** All the distributions are highly similar to those of ECFP4 shown in Figure 6.


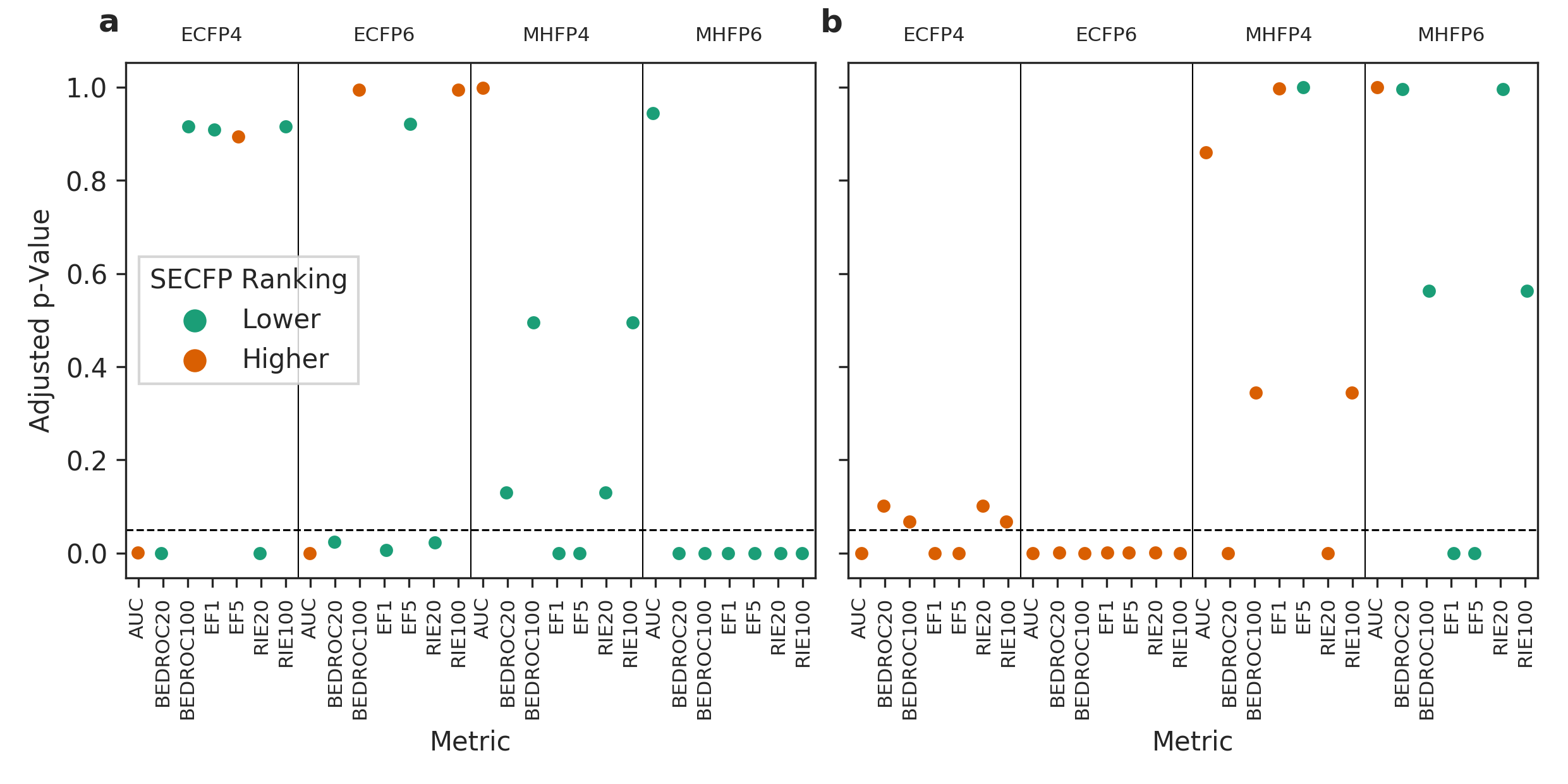


**Figure S9 Pairwise post-hoc Friedman tests of the average rank of SECFP4/6.** Statistical tests were run as part of the benchmark and visualized for easier comprehension. (a) Relative ranking and p-values of 2,048-D SECFP4 compared to ECFP4/6 and MHFP4/6 (all 2,048-D). (b) Relative ranking and p-values of 2,048-D SECFP6 compared to ECFP4/6 and MHFP4/6 (all 2,048-D). Orange color corresponds to SECFP4/6 being ranked higher than the other fingerprint, while green color indicates a lower ranking. P-values below 0.05 (dashed horizontal line) indicate significance.


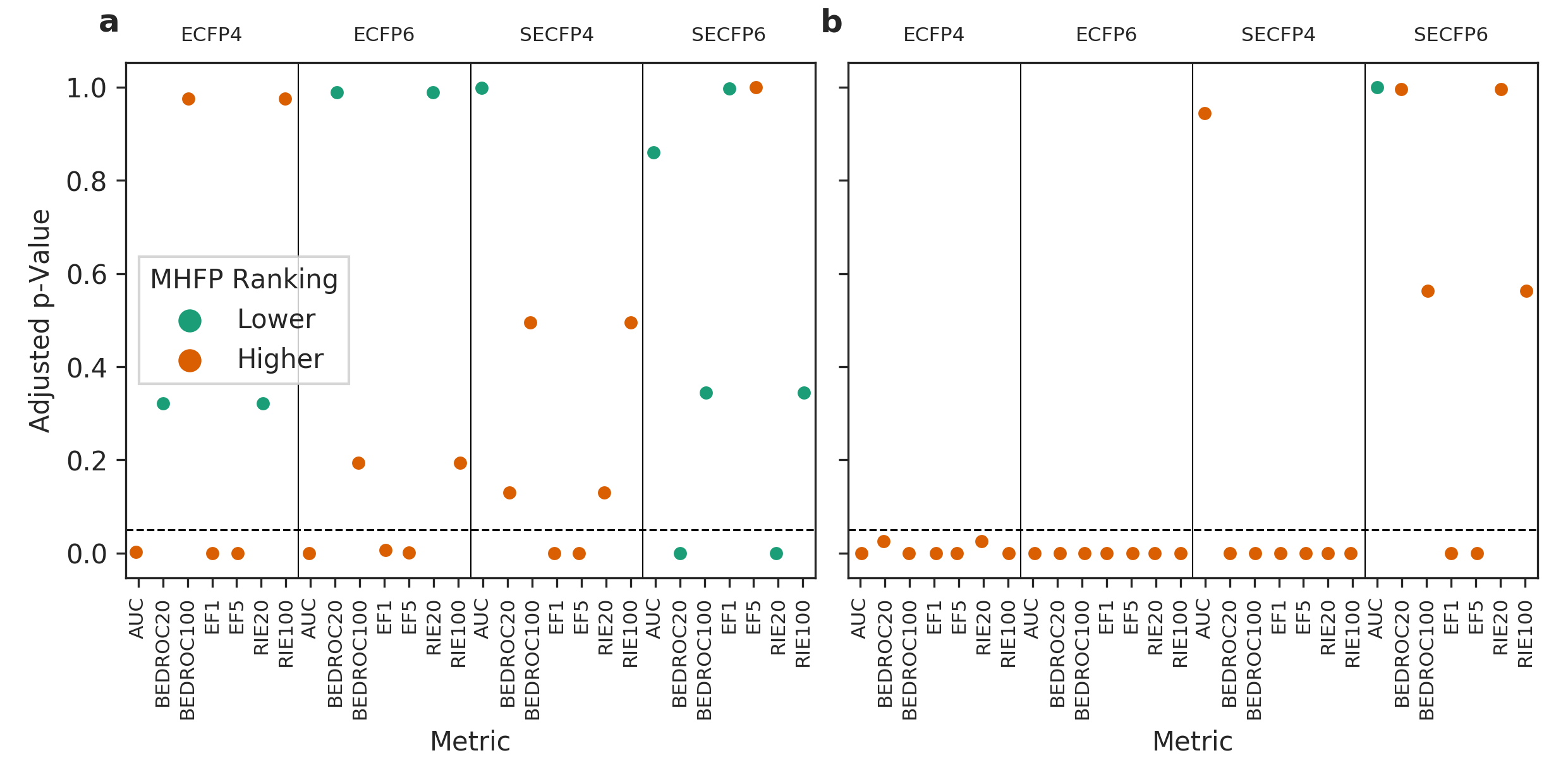


**Figure S10 Pairwise post-hoc Friedman tests of the average rank of MHFP4/6.** Statistical tests were run as part of the benchmark and visualized for easier comprehension. (a) Relative ranking and p-values of 2,048-D MHFP4 compared to ECFP4/6 and SECFP4/6 (all 2,048-D). (b) Relative ranking and p-values of 2,048-D MHFP6 compared to ECFP4/6 and SECFP4/6 (all 2,048-D). Orange color corresponds to MHFP4/6 being ranked higher than the other fingerprint, while green color indicates a lower ranking. P-values below 0.05 (dashed horizontal line) indicate significance.
